# Productivity impact of headache on a heavy-manufacturing workforce in Turkey

**DOI:** 10.1186/1129-2377-14-88

**Published:** 2013-10-30

**Authors:** Macit H Selekler, Gürsel Gökmen, Timothy J Steiner

**Affiliations:** 1 Department of Neurology, Kocaeli University Medical Faculty, İzmit, Kocaeli, Turkey; 2 Company Health Services, Ford Otomotiv Sanayi AŞ, Gölcük, Kocaeli, Turkey; 3 Department of Neuroscience, Norwegian University of Science and Technology, Trondheim, Norway; 4 Department of Neuroscience, Imperial College London, London, UK

**Keywords:** Headache disorders, Burden, Cost, Lost productivity, Work impact, Global Campaign against Headache

## Abstract

**Background:**

Headache disorders cause substantial productivity losses through absenteeism and impaired effectiveness at work (presenteeism). We measured productivity losses from both causes at a heavy-manufacturing company with a largely male workforce in north-western Turkey.

**Methods:**

We used the HALT Index as the survey instrument. We first assessed productivity losses by surveying the entire workforce. Because we anticipated much non-participation, we also applied HALT at the annual health-checks provided to all employees by the company’s on-site health clinic.

**Results:**

Mean age of the workforce (N = 7,200) was 31 yr. About two thirds (90% male) were manual workers rotating weekly through early, late and night shifts. One third (50% male) were clerical/managerial, working a standard 5-day week. In the first assessment, 3,939 questionnaires (54.7%) were returned with usable data. In the previous 3 months, absenteeism of ≥1 day was reported by 360 respondents (9.1%), of whom 4 (0.10%) recorded ≥45 days (average per worker: 0.92 days/yr). Presenteeism equivalent to ≥1 day’s absence was reported by 1,187 respondents (29.4%) (average per worker: 6.0 days/yr). We estimated that 23,519 days/yr were lost in total among respondents (2.3% of workforce capacity). In the first 6 months of annual health-checks, 2,691 employees (37.4%) attended (94.4% male). Absenteeism was reported by 40 (1.5%), with 74 days lost, presenteeism by 348 (12.9%), with 1,240 days lost. We estimated that, altogether, 41,771 man-days/yr were lost in the entire workforce (2.4% of capacity; 94% due to presenteeism), closely matching the earlier estimate. A small minority (5.7%) of those with headache, who were only 2.5% of the workforce, accounted for >45% of presenteeism-related lost productivity.

**Conclusion:**

The high productivity losses in a largely male workforce were surprising. Possible factors were the nature of the work – manual labour for two thirds, often heavy – and the recurring schedule disturbances of shift-work. There was a highly-disabled minority.

## Background

Headache disorders are common, affecting men, women and children in all parts of the world [1-4]. In Turkey, a recent nationwide survey found that almost 50% of the adult population had an active headache disorder and 3% were affected by headache on ≥15 days/month [5].

Headache disorders, especially migraine, impose symptom burden and disability. They lead to substantial demand for health care, which is poorly met [3]. They are most troublesome during the productive years of adulthood. In a United Kingdom survey [6], the reported loss of work-time due to migraine alone was 5.7 days per year per person affected, considerably skewed towards a very disabled small minority. From this it can be inferred that, in a mixed male/female workforce of 10,000 in the UK, of whom 15% are expected to have migraine, over 30 employees will be absent every working day. This estimate takes no account of reduced effectiveness in those at work despite being in a migraine attack (presenteeism). Neither does it include losses attributable to other headache disorders than migraine, among which those characterized by headache on ≥15 days/month are of considerable importance in this regard. These other disorders may, between them, multiply the losses two- or threefold, so that headache continuously reduces workforce capacity by >1%. The recent Eurolight project, a survey conducted in nine countries of the European Union, estimated societal losses attributable to all headache disorders (direct and indirect costs) at well over €100 billion per year [7], with more than 90% attributable to lost productivity. The findings indicated a workforce capacity reduction of at least 0.7% attributable to all headache disorders. Importantly, this estimate was discounted by 72%, the proportion who asserted that they could, and generally did, “make up” time later. It is questionable how reliable or realistic such assertions are, and in a manufacturing industry there may be no opportunity for restitution in this way. Furthermore, when an employee is a team member or, worse, a team leader, a whole group may become dysfunctional through the unexpected absence of that one person. Because of these factors, and also because conservative assumptions were built into the analytical model of Eurolight [7], a more correct estimate of workforce capacity reduction may be two or three times higher (*ie*, 1-2%), in line with the UK estimate [6].

Productivity losses from headache disorders are subject not only to their prevalence but also to culture, employment levels and, in individual cases, the nature of the particular job. Extrapolation from these findings to Turkey, and to specific employment settings within Turkey, may not be reliable. It is likely, nonetheless, that losses of considerable magnitude exist. In preparation for an interventional study to establish whether productive time losses would be recoverable through the provision of effective health care for headache (a project within the Global Campaign against Headache [8,9]), we undertook a study of headache-attributed time loss in the workforce of Ford Otomotiv Sanayi AŞ (FO), a vehicle manufacturing company in north-western Turkey.

At its manufacturing site in Gölcük, FO has a workforce of 7,200 employees. Their mean age is 31 years. Approximately two thirds (of whom 90% are male) are manual workers who rotate weekly through early, late and night shifts, each of 8 hours. One-third (50% male) are clerical or managerial, working a standard 5-day week, 8 am to 6 pm.

Ford Otosan maintains sickness-absence records for its employees, which do not, however, include reasons for absence. The company provides an on-site health clinic at each site, staffed *inter alia* by primary health-care physicians and nurses and providing care of day-to-day ailments. As a service to its employees, these clinics also carry out and record annual health-checks. These are offered, on a rotating basis to about 10% of the workforce each month.

Few of those with headache would be already receiving effective care, because headache services are not well established in primary care in Turkey. Although excellent care is available nearby from the University of Kocaeli, this is a tertiary-care facility to which only limited numbers have access (about 60 new headache patients are seen per month from the local population of 1.5 million, representing <0.25% of predicted need).

## Methods

### Ethics

Since this was the necessary first step in a project aimed specifically at service improvement, it fell outside the scope of research ethics review. While it involved the scrutiny of company medical records, this was done only by clinical staff directly involved in the employees’ medical care. It also involved, within the company, access to absenteeism records held legitimately by the employer.

Data-protection legislation was complied with. No personal information derived for the project passed beyond the confines of the health clinic without first being made anonymous.

### Lost productivity assessment

We used the HALT Index [10], translated into Turkish language, as the survey instrument. This instrument, a close derivative of the MIDAS questionnaire [11], discovers the existence of headache as a health problem in the respondent through enquiry into headache-attributed lost time. The first two questions ask about absenteeism due to headache and reduced productivity whilst at work with headache. To estimate total productive time lost per employee, we added days wholly lost through absenteeism and days of <50% productivity (presenteeism). We ignored headache-affected days in which productivity remained >50% by way of counterbalance. This is the principle behind the MIDAS questionnaire [11].

For the individual patient in a therapeutic encounter, the HALT Index, like MIDAS [11], counts days affected by headache in the previous 3 months (HALT-90). This is a compromise: it balances the need to reflect the illness over time in an individual against the considerable problems of recall bias. In a population measure using a large sample, there is no need to reflect the states of individuals, and therefore HALT can be applied, more reliably, over a shorter time of 1 month (HALT-30).

We made an initial assessment of productivity losses by surveying the entire workforce at Gölcük (N = 7,200). This survey invited anonymous returns so that employees might perceive no threat from the enquiry. HALT-90 was distributed to team leaders of every section of the factory, who, in turn, handed the questionnaires to all employees working with them. After completion, the questionnaires were returned via the team leaders to the on-site health clinic.

Despite maintaining anonymity, we anticipated a high level of non-participation in this survey, and were concerned about potential bias. Therefore, we commenced a more direct assessment. Over 6 months, HALT-30 was given to every employee during his or her annual health-check, along with the clinic’s own materials related to the health-check. These forms, completed prior to the health-check, were collected by the supervising physician. As this enquiry was in the context of individual health care, responses could be coupled with health records held by the medical staff. They could also be coupled with health and sickness-related absenteeism records, but we did not do this.

### Data entry and management

Questionnaires from the first survey, on its completion, were divided between three health technicians, who transferred the data to Excel sheets. In the second survey, questionnaires were collected day by day during the annual health-checks and data transferred to Excel by one health technician. Subsequently, all Excel sheets were converted to SPSS (Statistical Package for Social Sciences for Windows version 15.0).

### Statistics

We used SPSS for descriptive statistical analyses, calculating (as appropriate) mean, standard deviation (SD), median and inter-quartile range (IQR).

## Results

Of 7,200 questionnaires distributed in the first assessment, 4,033 were returned, 3,939 (54.7%) with usable data. Because the biases introduced by self-selecting participation in this survey were uncertain, we based the following estimates on denominators both of 7,200 (the entire workforce) and of 3,939 (the responding sample), to yield ranges; the lower limits of these ranges were conservative, while true estimates would fall somewhere within the ranges.

**Figure 1:**
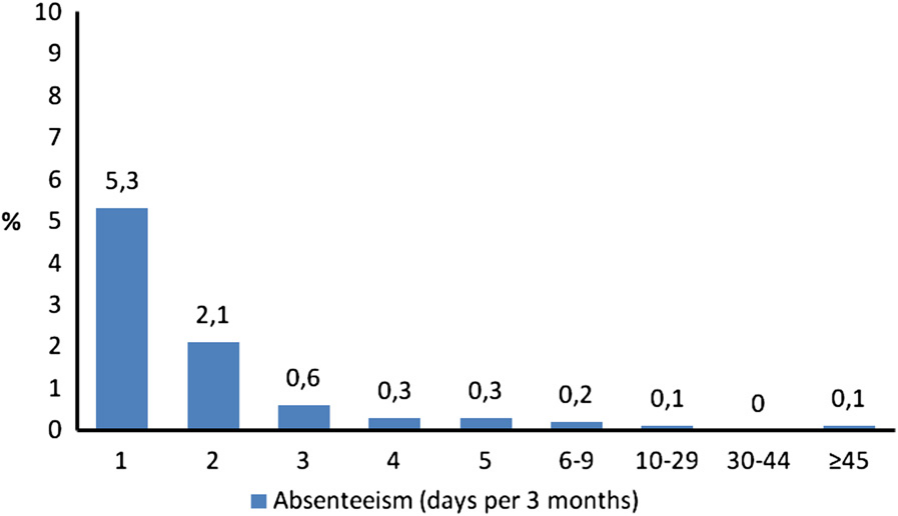
Distribution of absenteeism in the preceding 3 months among the workforce sample (N = 3,939) in the first survey.

Absenteeism of at least 1 day was reported in the previous 3 months by 360 respondents (representing 5.0-9.1% of the workforce). Of these, four (0.06-0.10%) reported absences of ≥45 days, providing evidence of a highly-disabled small minority in a non-normal distribution (Figure 1). Total absenteeism for 3 months was 920 days. The mean absenteeism per person among the 360 was 2.55 ± 6.17 (median: 1; inter-quartile range: 1–2). Average absenteeism per worker in the entire workforce (*ie*, including those with and without headache) was 0.51-0.93 days per year.

**Figure 2:**
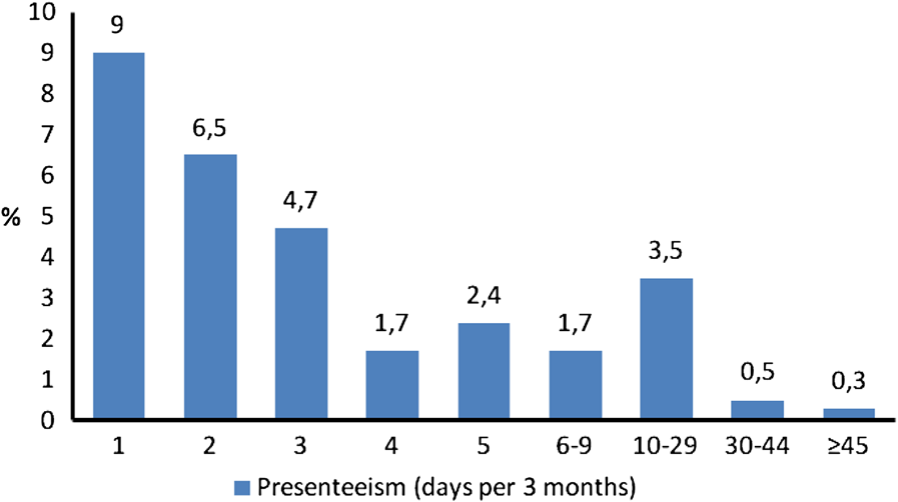
Distribution of presenteeism in the preceding 3 months among the workforce sample (N = 3,939) in the first survey.

Lost productivity through having headache while at work (presenteeism) and equivalent to ≥1 day’s absence in the previous 3 months was reported by 1,187 (16.5-30.1%) of the workforce. Of these, 13 (0.18-0.33%) reported lost productivity equivalent to absence on ≥45 days, similarly reflecting a highly-disabled minority (Figure 2). Total presenteeism for 3 months was 5,873 days. The mean presenteeism per person among the 1,187 was 4.95 ± 9.07 (median: 2; IQR: 1–5). Average presenteeism-related lost productivity per worker in the entire workforce (*ie*, including those with and without headache) was 3.3-6.0 days per year.

We detected overlap between the 360 reporting absenteeism and the 1,187 reporting presenteeism, and suspected a degree of double counting. Therefore, in a re-analysis, rather than add the two we took only the higher of the two reported numbers (that is, we assumed, whenever absenteeism and presenteeism were both reported, that there was always double counting and that whichever was the higher number of days subsumed the lower). On this conservative basis we estimated that at least 23,492 days’ productivity was lost per year at Gölcük (1.3-2.3% of workforce capacity, assuming employees worked on average for 5 days per week and 48 weeks per year).

**Figure 3:**
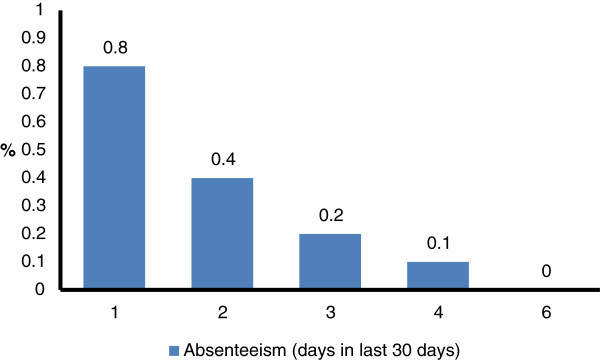
Distribution of absenteeism in the preceding 30 days among the workforce sample (N = 2,691) in the second survey.

In the 6 months of annual health-checks, a total of 2,691 employees (37.4% of the workforce) attended, of whom 2,541 (94.4%) were male and 150 (5.6%) female. Mean age was 32.7 ± 5.4 years (range 20–55). In the months April to September, 459, 793, 270, 347, 107 and 715 respectively were seen (in the summer months, periodic examinations are reduced, and the factory shuts down during August). Of these, 1,162 people (43.2%) reported headache on at least 1 day in the previous month. Absenteeism was reported by 40 (3.4% of those reporting headache and 1.48% of the total sample), who recorded 74 days lost in total, a mean per person of 1.85 ± 1.17 (median: 1; IQR: 1–2) (Figure 3).

**Figure 4:**
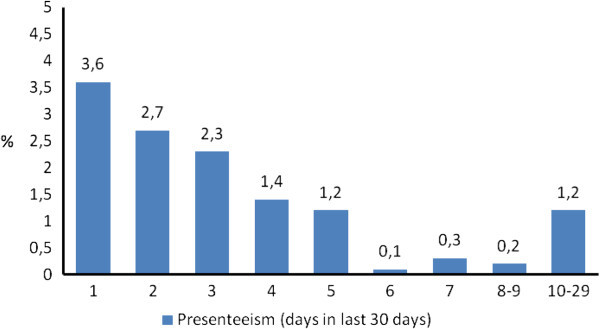
Distribution of presenteeism in the preceding 30 days among the workforce sample (N = 2,691) in the second survey.

Presenteeism equivalent to at least 1 day lost was reported by 348 (29.9% of those reporting headache and 12.9% of the total sample), who recorded 1,256 days lost in total (mean per person: 3.61 ± 3.51; median: 3; inter-quartile range 1–4) (Figure 4). Lost days (per month) were altogether 1,330, by far the greater part (94%) attributed to presenteeism.

Since the general pattern reflected far fewer days of absenteeism than of presenteeism, we suspected double counting in nine questionnaires recording the same number of days for each. We ignored presenteeism in such cases, which was conservative. Effectively this reduced the number reporting presenteeism to 339 (12.6% of the sample) and the days affected to 1,227. The revised total of days lost was 1,301. The ratio of presenteeism to absenteeism was 16.6:1.

On the basis of the annual health-checks, we estimated that productivity equivalent to 41,771 man-days was lost per year at Gölcük (2.4% of workforce capacity), matching the upper limit of the estimate from the first survey.

Probability of presenteeism was strongly driven by reported headache frequency. Those with headache on 9–14 days/month (n = 39; 3.3% of those with any headache) lost 248 days (20.2% of all lost days); those with headache on ≥15 days/month (n = 27; 2.3% of those with any headache) lost 307 days (25.0% of all lost days). In other words, 5.7% of those with headache, who were only 2.5% of the workforce, accounted for >45% of presenteeism-related lost productivity.

Probabilities of absenteeism and presenteeism were not correlated: 35 people reporting absenteeism had only 23 days of presenteeism, while the remaining five recorded 65 days of presenteeism. Thus 87.5% of people losing days through absenteeism contributed little to lost days through presenteeism.

## Discussion

The major strength of this study was the use of two distinct survey methods, each arriving at the same estimate. The particular strengths of the second part of the study, based on annual health-checks, were first that these take place rotationally, with no selection by the employer, and second that they are offered in a health-care context, for the employees’ benefit, with very high uptake. Both these factors went a long way to eliminating participation bias. It was an unavoidable limitation that employees’ reports were not objectively verifiable; in a health-care setting there are no reasons to provide misleading responses, but this does not guarantee complete veracity. Additionally, estimates of presenteeism are inevitably both subjective and approximate, but use of HALT-30 rather than HALT-90 at least was expected to reduce recall bias, another major potential source of error.

From the annual health-checks in 2,691 employees, we recorded a 1-month prevalence of headache of 43.2%. It is difficult to relate this to 1-year prevalence, although the latter must be higher, including people experiencing headache less than the median frequency of once a month. The finding is certainly compatible with the recent nationwide survey in Turkey, which recorded a 1-year prevalence of almost 50% [5]. Most of the workforce at FO were male, who might have been expected to have less headache than the population average, but they were of an age (mean 32.7 years) at which headache disorders – migraine in particular – tend to be troublesome.

Work loss attributable to headache has three distinguishable components. Intermittent absence is an expected consequence of a recurrent painful disorder, visible and within limits credible to employers; and it is relatively easy to measure. Reduced productivity whilst at work despite headache (presenteeism) is less visible and much more difficult to measure. Complete inability to work gainfully is not apparent to employers, but a significant drain on social security budgets. It is a relatively uncommon consequence of headache alone; when it happens, it is usually associated with frequently-recurring or unremitting intractable headache, often with co-morbidities. This study focused on a group of people in paid employment, and addressed the first two components of work loss.

The biases introduced by selective participation in the first survey were uncertain, but we thought it likely that those troubled by headache would have been among the responders. We calculated ranges for each estimate based on two denominators – those responding, and the entire workforce – but, if our supposition was correct, the lower limit of each range (higher denominator) would better apply. In fact, for lost workforce capacity attributable to headache, the range was 1.3-2.3% while the corresponding estimate of 2.4% from the annual health-checks was in very close agreement with the *upper* limit. We expected the latter estimate to be more robust, for the reasons given earlier. Three factors may have been relevant. First, in the initial survey the supposition of preferential self-selection of those troubled by headache might have been incorrect: it seemed likely, but had no empirical basis. If, in fact, the 54.7% usable returned questionnaires were a perfectly representative sample, then the two surveys were in complete accord. Second, the initial survey might well have been perceived as a management-led initiative and, therefore, with some suspicion. The expected consequence would be under-reporting. The health-care setting of the second survey addressed this. Third, as noted above, we reduced the estimate from the initial survey to eliminate suspected double-counting, and this might have been over-conservative. We therefore prefer the estimate yielded by the annual health-checks. There is, perhaps, a methodological lesson arising from this, of which future employee surveys should take due note.

This said, the estimate is well above expectation informed by studies in Europe [7], suggesting a higher prevalence of disabling headache in Turkey than the European average [2]. The recent survey in Turkey reported a 1-year prevalence of migraine of 28.8% (definite plus probable) [5], which clearly supports this. What is surprising is this amount of loss in a largely male workforce. Age (mean 32.7 years) has already been noted as a factor; others may be the nature of the work – manual labour for two thirds, often heavy – and, maybe more contributory, the fact that these workers rotate weekly through early, late and night shifts (schedule disturbances have long been recognized as a potent migraine trigger [12]).

The high presenteeism to absenteeism ratio (16.6:1) may appear remarkable. From employee records, the average absence rate for the entire workforce in 2010 (not attributable to any specific illness) was 4.54 days per person, a workforce capacity reduction of 1.7%. The importance of this finding is that presenteeism is largely hidden to employers, who, on this evidence, are led to believe that lost productivity from headache is unimportant since they see less than 6% of it. In the face of disabling illness, many factors contribute to presenteeism rather than absenteeism [13]. We did not look at these, because they were not central to our purpose of measuring and reporting productivity impact – a major part of the burden of headache [7,14] – as opposed to explaining it. Future research may need to examine these factors, and will have to do so in various settings, especially because this high ratio has not been seen in headache surveys elsewhere [7,14]. We can meanwhile offer one simple explanation, which is that the unemployment situation in Turkey makes employees reluctant to be absent.

An interesting finding to emerge, and perhaps related to this, was that 87.5% of people losing days through absenteeism contributed little to lost days through presenteeism. It is worth noting that HALT does not measure *disability* but behavioural response to impairment: unless disability is severe, absenteeism to some extent reflects choice. On the other hand, maybe these 35 people were affected in a particular way, necessitating absenteeism. Nausea and vomiting, among the symptoms of migraine, make it particularly difficult to travel to and be at work.

A small minority (5.7%) of those with headache (2.5% of the workforce) accounted for >45% of presenteeism-related lost productivity. This operation of the Pareto principle has been reported elsewhere. In the UK survey [6], lost work-time due to migraine, averaging 5.7 days per year per person affected, was considerably skewed towards a very disabled small minority. In a Swedish survey, 3-4% of the population had most of the burden of migraine [15]. Although they have not been well studied epidemiologically, the group of disorders characterized by headache on ≥15 days/month, which include medication-overuse headache and are estimated to affect 3% of adults in Turkey [5], undoubtedly impose the highest individual burden [7]. There is an important implication in this for intervention: if the objective is to reduce lost productivity, health care would most efficiently be aimed at the people who make up this disabled minority. This presupposes, of course, that these people are treatable. And in pursuit of this objective, the less pressing but still significant needs of the majority should not be overlooked.

## Conclusions

Estimates of productivity losses were well above expectation informed by studies in Europe, particularly surprising in a largely male workforce but perhaps explicable by a high prevalence of headache in Turkey, the nature of the work and the schedule disturbances of shift-work. As seen elsewhere, there was a highly-disabled minority.

Although this study did not have this primary purpose (it was a base-line assessment prior to an interventional study), it adds to the now incontrovertible evidence that headache disorders are hugely costly to national economies [3,6,7,14] (Andrée C, Steiner TJ, Barré J et al. Headache yesterday in Europe. submitted for publication). How much longer will it be before this is politically recognized [3,16,17]?

## Competing interests

HMS declares no conflicts of interest. GG is an employee of Ford Otomotiv Sanayi AŞ. Any opinions expressed herein are his own and not those of the company. The management of Ford Otomotiv gave their full cooperation to the study, but were not involved in its design and conduct, and had no input into this report. TJS is a director of *Lifting The Burden*.

## Authors’ contributions

HMS contributions were data collection, statistical analysis and writing the manuscript. GG had contribution in data collection and statistical calculations. TJS had planned to study and contributed to statistical analysis and writing the manuscript. All authors read and approved the final manuscript.
